# Renal Precision Medicine in Neonates and Acute Kidney Injury: How to Convert a Cloud of Creatinine Observations to Support Clinical Decisions

**DOI:** 10.3389/fped.2020.00366

**Published:** 2020-07-28

**Authors:** Karel Allegaert, Anne Smits, Tamara van Donge, John van den Anker, Kosmas Sarafidis, Elena Levtchenko, Djalila Mekahli

**Affiliations:** ^1^Department of Development and Regeneration, KU Leuven, Leuven, Belgium; ^2^Department of Pharmacy and Pharmaceutical Sciences, KU Leuven, Leuven, Belgium; ^3^Department of Clinical Pharmacy, Erasmus MC, Rotterdam, Netherlands; ^4^Neonatal Intensive Care Unit, University Hospitals Leuven, Leuven, Belgium; ^5^Pediatric Pharmacology and Pharmacometrics, University of Basel Children's Hospital (UKBB), University of Basel, Basel, Switzerland; ^6^Division of Clinical Pharmacology, Children's National Hospital, Washington, DC, United States; ^7^Intensive Care and Department of Pediatric Surgery, Erasmus MC Sophia Children's Hospital, Rotterdam, Netherlands; ^8^First Department of Neonatology, School of Medicine, Aristotle University of Thessaloniki, Hippokrateion General Hospital, Thessaloniki, Greece; ^9^Department of Pediatric Nephrology and Organ Transplantation, Hospitals Leuven, Leuven, Belgium

**Keywords:** creatinine, Cystatin C, precision medicine, acute kidney injury, newborn, nephron number

## Abstract

Renal precision medicine in neonates is useful to support decision making on pharmacotherapy, signal detection of adverse (drug) events, and individual prediction of short- and long-term prognosis. To estimate kidney function or glomerular filtration rate (GFR), the most commonly measured and readily accessible biomarker is serum creatinine (S_cr_). However, there is extensive variability in S_cr_ observations and GFR estimates within the neonatal population, because of developmental physiology and superimposed pathology. Furthermore, assay related differences still matter for S_cr_, but also exist for Cystatin C. Observations in extreme low birth weight (ELBW) and term asphyxiated neonates will illustrate how renal precision medicine contributes to neonatal precision medicine. When the Kidney Disease Improving Global Outcome (KDIGO) definition of acute kidney injury (AKI) is used, this results in an incidence up to 50% in ELBW neonates, associated with increased mortality and morbidity. However, urine output criteria needed adaptations to broader time intervals or weight trends, while S_cr_ and its trends do not provide sufficient detail on kidney function between ELBW neonates. Instead, we suggest to use assay-specific centile S_cr_ values to better describe postnatal trends and have illustrated its relevance by quantifying an adverse drug event (ibuprofen) and by explaining individual amikacin clearance. Term asphyxiated neonates also commonly display AKI. While oliguria is a specific AKI indicator, the majority of term asphyxiated cases are non-oliguric. Asphyxia results in a clinical significant—commonly transient—mean GFR decrease (−50%) with a lower renal drug elimination. But there is still major (unexplained) inter-individual variability in GFR and subsequent renal drug elimination between these asphyxiated neonates. Recently, the Baby-NINJA (nephrotoxic injury negated by just-in-time action) study provided evidence on the concept that a focus on nephrotoxic injury negation has a significant impact on AKI incidence and severity. It is hereby important to realize that follow-up should not be discontinued at discharge, as there are concerns about long-term renal outcome. These illustrations suggest that integration of renal (patho)physiology into neonatal precision medicine are an important tool to improve contemporary neonatal care, not only for the short-term but also with a positive health impact throughout life.

## Introduction

Precision medicine is defined as a structured approach to treat or prevent specific diseases based on the inter-individual variability in genes, physiology, and environment. This includes exploration of novel research approaches to improve the use of available information to support decision making about pharmacotherapy, signal detection of adverse (drug) events, or to improve individual prediction of short- and long-term prognosis. Neonatal renal precision medicine depends on the availability of reference intervals for any renal biomarker to support clinical decision making, tailor therapy, or support prognosis. This is still a major limitation, highlighted in the International Neonatal Consortium (INC) paper on safety, dosing, and pharmaceutical quality of medical products in neonates and during development of the neonatal adverse event severity scale. Severity grading for lab values—including kidney function—was omitted until reference values became available (1, 2).

**Figure 1:**
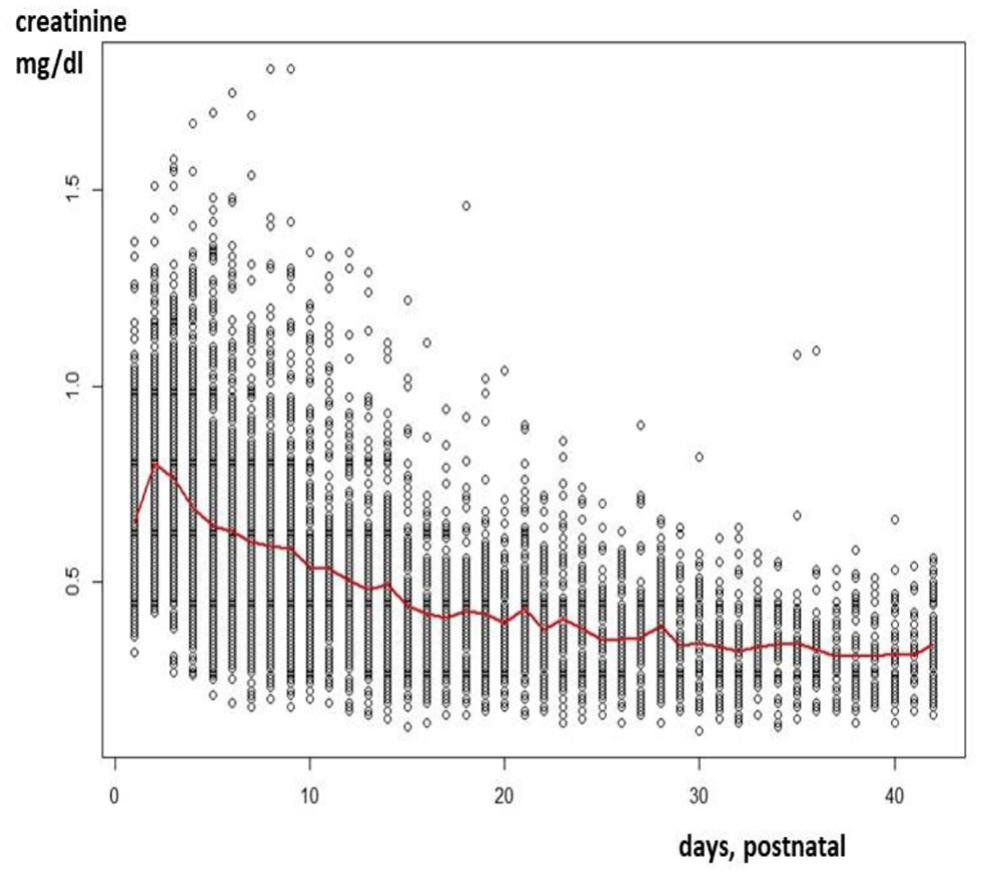
Creatinine values (enzymatic assay) as observed in a cohort of 1,140 neonates (gestational age 23–42 weeks) in the first 42 days of postnatal life (3).

Since maturational physiological changes are most prominent in early infancy, variability is their key feature. This is reflected in extensive inter- and intra-individual variability in serum creatinine (S_cr_), resulting in a cloud instead of extractable and interpretable information for clinicians. This “cloud” is illustrated in Figure 1, after plotting S_cr_ observations (enzymatic assay) in (pre)term neonates collected in one Neonatal Intensive Care Unit (NICU) in the first 42 postnatal days of life (3). This reflects a pattern with an initial increase and subsequent decrease during postnatal life. As relevant, there is about a 4-fold difference in S_cr_ observed for all consecutive days, so that improved understanding on reference values is needed to attain precision medicine.

This S_cr_ variability is partly explained by maturational changes (e.g., birth weight, gestational age [GA], postnatal age) and non-maturational changes related to pathophysiology, e.g., perinatal asphyxia, co-medication, congenital anomalies of the kidney and urinary track (CAKUT), cardiac surgery with bypass, or extra-corporeal membrane oxygenation (4, 5). Postnatal kidney adaptation is proportional to the nephron number (GA driven) and renal perfusion (postnatal adaptation, mean arterial blood pressure) (6, 7). Nephrogenesis evolves as branching morphogenesis, similar to lung, pancreas, vascular tree, or retina. Neonates <36 GA weeks are still in active nephrogenesis and thus have an increased risk for decreased nephron endowment with lifelong impact (8). The main determinants of the increase in renal function in early life are circulatory changes, driven by the increase in proportional renal blood flow to cardiac output, from 2 to 25%. This increased renal blood flow is combined with dilation of the efferent and constriction of the afferent arterioli. This explains the significant impact of non-steroidal anti-inflammatory drugs (NSAIDs, −20 up to −40% of the glomerular filtration rate [GFR] in ELBW neonates, depending on the type and dose) or asphyxia (up to −40 to −50% of GFR).

## Creatinine and Cystatin C as Biomarkers of GFR in Newborns

S_cr_ is the most commonly measured biomarker to estimate kidney function or GFR (creatinine clearance). However, before S_cr_ can be used to estimate renal elimination capacity in neonates, issues that should be considered relate to physiology (renal tubular transport, hydration, muscle mass) and measurement (assay validity).

Creatinine at birth does not yet reflect neonatal but maternal S_cr_ levels. Because of passive tubular back leak instead of active secretion, creatinine clearance does not yet fully reflect GFR. In contrast to later life, where creatinine clearance somewhat overestimates GFR due to active tubular secretion mediated by Organic Cation Transporter 2 (OCT-2), passive back leak occurs in early neonatal life (9, 10). Hydration is another issue, as early neonatal life is associated with weight reduction due to free water loss, usually associated with a sodium increase. Creatinine is a low molecular weight (133 g/mol) molecule produced by muscle catabolism (creatine to creatinine), reflecting muscle mass. As estimated by creatinine excretion in urine, muscle mass increased from 12% of birth weight at 25 weeks to 19% at 34 GA weeks and 24% at term (11). In contrast, the proportional muscle mass was estimated to remain stable (22–30%) without trend related to GA in autopsy findings (12).

S_cr_ values also depend on the assay, as the Jaffe assay is affected by specific constituents of neonatal serum like bilirubin or albumin concentrations. Harmonization through isotope dilution mass spectrometry (IDMS) traceability has reduced, but not eliminated, this inter-assay variability (9, 13). S_cr_ measurements can subsequently be converted to estimated GFR, using the Schwartz formula (eGFR = *k* [*L*/S_cr_], S_cr_ = μmol/l; *k* = 0.34 in preterm, 0.45 in term infants, *L* = length, cm). One should hereby be aware that this Schwartz formula has initially been validated (to inulin clearance) with the original, non-compensated Jaffe assay (9). Furthermore, length measurement has limitations in neonates (14). While lower *k*-values have been suggested when enzymatic assays are used, these studies have not included (pre)term neonates and infants (9). Recently, an eGFR specific to (pre)term neonates (median age 3 days postnatal age, compensated Jaffe) was suggested (eGFR= 2.32 × [weight (g)^0.64^/S_cr_ (μmol/l)^0.62^]) following validation with inulin clearance (21.54 [SD 10.09] ml/min/1.73 m^2^). This formula performed somewhat better compared to the original neonatal Schwartz formula (15).

**Figure 2:**
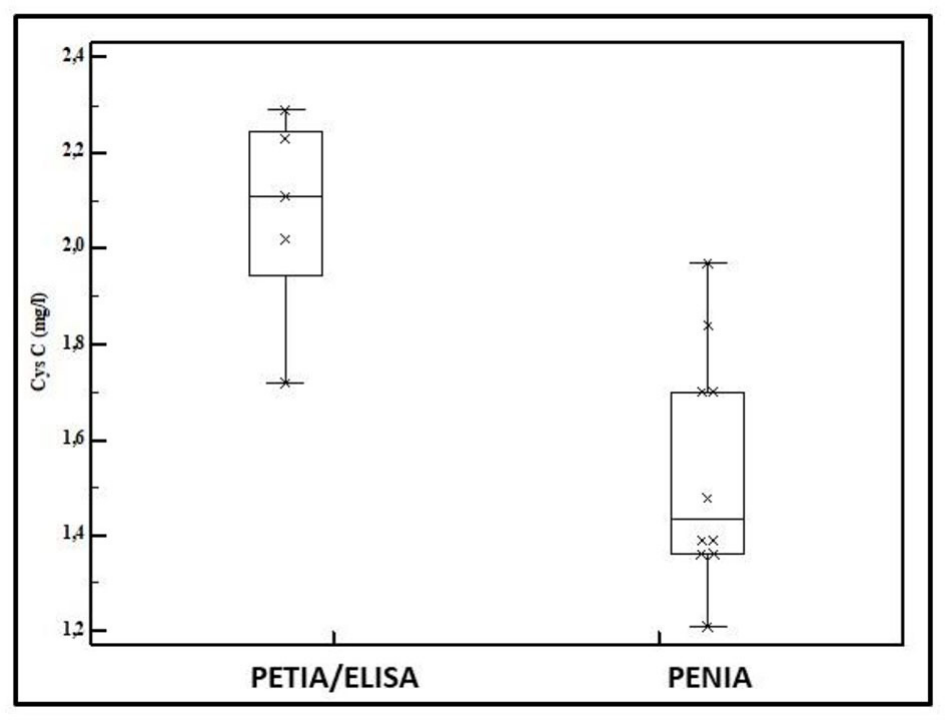
Mean Cystatin C values in umbilical cord blood as reported in 15 cohorts of healthy term neonates (2 enzymatic immuno-assay [ELISA], three particle enhanced turbimetric immuno-assay [PETIA], and 10 particle enhanced nephelometric immuno-assay [PENIA]) (16–22).

Cystatin C is an alternative to S_cr_ to assess eGFR and is considered to be a more sensitive indicator for minor GFR changes. Cystatin C is a 130 amino acids containing small protein, generated by any cell with elimination by GFR. Reference values have been suggested, but Cystatin C also has assay-related issues like S_cr_. Figure 2 reflects assay-specific differences in mean umbilical cord blood Cystatin C values reported in 15 cohorts of healthy term neonates. In these 15 cohorts, Cystatin C was quantified by enzymatic immuno-assays (ELISA, *n* = 2), particle enhanced turbimetric immuno-assays (PETIA, *n* = 3), or particle enhanced nephelometric immuno-assays (PENIA, *n* = 10) (16–22). Since 2010, certified reference material for Cystatin C assay standardization (IDMS) has been available, but measurement bias still exists (23). This is relevant, since the majority of studies with Cystatin C in neonates were conducted in single units or with one assay. Within this setting, Cystatin C values are associated with maturational covariates (age, weight) or perinatal diseases, like congenital renal anomalies, sepsis, and septic shock, respiratory distress, hypotension, transient tachypnea of the newborn, or perinatal asphyxia, serving as a more sensitive indicator for renal dysfunction (16, 24, 25). However, clinicians should be cautious in extracting absolute values as reported for subsequent use in their specific setting as assay-related issues may exist. Of specific relevance to neonates, steroid administration or hypothyroidism may also affect Cystatin C (16, 26). Finally, Cystatin C was not retained in the recently published eGFR formula specific to (pre)term neonates (15).

We therefore suggest development of age-dependent, assay specific S_cr_ centiles to support clinical decisions and precision pharmacotherapy. We will focus on ELBW (<1,000 g) infants and on term neonates with perinatal asphyxia undergoing whole body hypothermia (WBH) to illustrate how a “S_cr_ cloud” can be converted into a clinical decision tool. With these examples, we will illustrate how renal precision medicine is a crucial part of modern neonatal care. A similar approach can be considered for other subcategories like CAKUT newborns or neonates in need of cardiac bypass or extracorporeal membrane oxygenation.

### Key Messages

- There is extensive variability in S_cr_ and eGFR (Schwartz formula, Wilhelm-Bals formula) within the neonatal population, driven by (patho)physiology.
- Assay related differences exist for S_cr_ and Cystatin C.
- We suggest to develop age-dependent, assay specific S_cr_ centiles to support neonatal precision medicine.

## Renal Function, AKI, and Precision Medicine in ELBW Infants

Almost a decade ago, Jetton and Askenazi (27) suggested an AKI definition (neonatal modified KDIGO) specific for use in neonates. In essence, the definition is based on trends (increase) *in S*_cr_ and *in urine output* to result in staging (stage 0–3) (Table 1). This definition was endorsed by a National Institute of Diabetes, Digestive, and Kidney (NIDDK) Diseases workshop. The attendees hereby concluded that this definition offered a reasonable starting point, but that further evaluation was needed (30, 31). In this context, a study protocol (Assessment of Worldwide Acute Kidney Injury Epidemiology in Neonates, AWAKEN) was put forward to assess its applicability and to report on neonatal AKI epidemiology (28).

**Table 1:** Definition of neonatal Acute Kidney Injury (AKI) by serum creatinine and urine output (28, 29).

**Stage**	**Serum creatinine (S_cr_)**	**Urine output**
0	No change in S_cr_ or ↑ <0.3 mg/dl	>1 ml/kg/h
1	S_cr_ ↑≥ 0.3 mg/dl within 48 h or S_cr_ ↑≥1.5–1.9 X vs. S_cr_^*^ within 7 days	>0.5 and ≤1 ml/kg/h
2	S_cr_ ↑≥ 2 to 2.9 vs. reference S_cr_^*^	>0.3 and ≤0.5 ml/kg/h
3	S_cr_ ↑≥ 3 X reference S_cr_^*^ or S_cr_ ≥2·5 mg/dl^**^or dialysis	≤0.3 ml/kg/h

* *Reference S_cr_ is the lowest prior S_cr_ measurement*;

** *a S_cr_ of 2.5 mg/dl in neonates reflects an estimated glomerular filtration rate <10 ml/min/1.73 m^2^*.

We highlight some results of this AWAKEN study specific to ELBW neonates to emphasize the limitations of the KDIGO definition: (*i*) the overall AKI incidence was 29.9%, but was 47.9% in preterm neonates (<29 weeks GA) (*limited discriminating power*); (*ii*) 8/24 of the contributing units still used a Jaffe assay (*relevant differences in absolute values not considered in the definition*); (*iii*) the median number of S_cr_ counts was ≤3 and ≤5/patient respectively in 10 and 15/24 of these units (*suggesting that there is not yet sufficient focus on renal function, even in AWAKEN units*) (*iv*) urine output has been quantified in 24 h intervals with 1 ml/kg/h as pivotal finding (*pragmatic, but not as suggested in the definition*, Table 1), (*v*) despite these limitations, the AKI stage predicted mortality (adjusted Odds Ratio 3.7) but not length of stay in <29 weeks neonates (29). In secondary analyses specific in the most immature cohorts, an increased risk for bronchopulmonary dysplasia was observed in 29–32 GA cases (adjusted Odds Ratio 4.2), but not in <29 GA neonates (32). Early (*within the first week of life*) caffeine administration was associated with reduced AKI incidence (number needed to treat = 4.3) and severity (33). This can be explained by the adenosine related effects on the glomerular vascular tone. Finally, AKI was not limited to early neonatal life but also occurred beyond day 7 (9%). Risk factors were the presence of a patent ductus arteriosus with or without NSAIDs exposure, necrotizing enterocolitis, and sepsis (34).

Although the current AKI definition may assist clinicians to recognize renal issues, there are still limitations to using this AKI tool for precision medicine in ELBW cases. As 47.9% of cases <29 weeks were classified as having AKI, the granularity needed for precision medicine is somewhat lost. The issues relate to both urine output and S_cr_ as biomarkers of kidney function and AKI.

Continuous quantification of urine output is a technical burden and is even more difficult in the most immature neonates, as catheterization is invasive while sequential diaper weight is hampered by evaporation (up to 80% weight losses after 2 h of a 5 ml portion added to a diaper exposed in an incubator or under a radiant warmer) (35). This was already acknowledged by the AWAKEN study, since urine output was quantified by 24 h increments with 12% missing observations for urine output (29). Fluid overload and daily weight balance (change% = current weight – birthweight/current weight) in the 1st week of life were used as alternative markers. Based on these markers, a higher positive peak in the 1st week of life and a positive fluid balance on day 7 were associated with mechanical ventilation (36).

S_cr_ itself is not an AKI biomarker, but rather an indicator of kidney function. As mentioned earlier, absolute values are affected by the assay (dependent on IDMS traceability), since Jaffe results are affected by some drugs and—more relevant to ELBW neonates—by bilirubin so that the median difference between the original Jaffe and an enzymatic assay is 0.12 to 0.27 mg/l, with always higher values for the Jaffe assay (37). Furthermore, the maturational S_cr_ changes over postnatal age are extensive in ELBW neonates. There is an initial increase to peak on day 3, with a subsequent slow decrease over postnatal age (38, 39). To further illustrate this, we have summarized the postnatal S_cr_ trends (10th, 25th, median, 75th, 90th, and 95th centile) over the first 28 days of life in an cohort of 217 ELBW cases (single unit, enzymatic assay, all exposed to caffeine) in Table 2 (38). If we focus on the median estimates, there is a clear increase from day 1 to 3 by 0.3 mg/dl, so that this median “normal” trend already qualifies for an AKI stage 1 classification. From a physiological point of view, it may even be reasonable to classify a relevant portion of ELBW as having AKI. However, by using a centile approach, more “granularity” in the data is provided to facilitate precision medicine.

**Table 2:** Centiles (10th−95th) of serum creatinine values (enzymatic assay) in a cohort of 217 extremely low birth weight (ELBW) infants in the first 28 days of postnatal age (38).

	**Day 1**	**Day 2**	**Day 3**	**Day 4**	**Day 5**	**Day 6**	**Day 7**	**Day 8**	**Day 9**	**Day 10**	**Day 11**	**Day 12**	**Day 13**	**Day 14**
**Samples**	206	190	205	198	182	171	145	157	140	128	117	112	92	133
10th	0.45	0.64	0.74	0.64	0.58	0.55	0.54	0.50	0.50	0.44	0.42	0.41	0.41	0.41
25th	0.52	0.75	0.81	0.76	0.7	0.67	0.64	0.60	0.57	0.55	0.51	0.50	0.47	0.48
50th	***0.605***	***0.86***	***0.91***	***0.88***	***0.84***	***0.80***	***0.75***	***0.74***	***0.70***	***0.65***	***0.64***	***0.60***	***0.59***	***0.57***
75th	0.74	0.95	1.03	1.03	0.97	0.94	0.89	0.88	0.83	0.79	0.79	0.76	0.70	0.66
90th	0.91	1.065	1.18	1.18	1.14	1.12	1.11	1.04	1.01	0.93	0.96	0.87	0.86	0.8
95th	0.98	1.16	1.22	1.29	1.25	1.28	1.17	1.12	1.15	1.05	1,03	0.92	0.96	0.85
	**Day 15**	**Day 16**	**Day 17**	**Day 18**	**Day 19**	**Day 20**	**Day 21**	**Day 22**	**Day 23**	**Day 24**	**Day 25**	**Day 26**	**Day 27**	**Day 28**
**Samples**	101	96	99	79	85	62	123	72	66	86	64	50	54	111
10th	0.36	0.36	0.37	0.35	0.38	0.37	0.36	0.37	0.36	0.35	0.33	0.34	0.32	0.32
25th	0.44	0.44	0.43	0.42	0.42	0.40	0.39	0.40	0.40	0.41	0.38	0.37	0.37	0.36
50th	***0.55***	***0.52***	***0.51***	***0.49***	***0.49***	***0.50***	***0.47***	***0.46***	***0.47***	***0.47***	***0.43***	***0.45***	***0.44***	***0.42***
75th	0.63	0.61	0.60	0.61	0.57	0.62	0.54	0.54	0.54	0.52	0.52	0.51	0.50	0.48
90th	0.81	0.69	0.71	0.73	0.66	0.68	0.62	0.68	0.64	0.64	0.58	0.59	0.70	0.55
95th	0.87	0.73	0.78	0.81	0.73	n.a.	0.68	0.72	n.a.	0.67	n.a.	n.a.	n.a.	0.59

*The numbers in bold and italic represent the 50th centile, or the median*.

**Figure 3:**
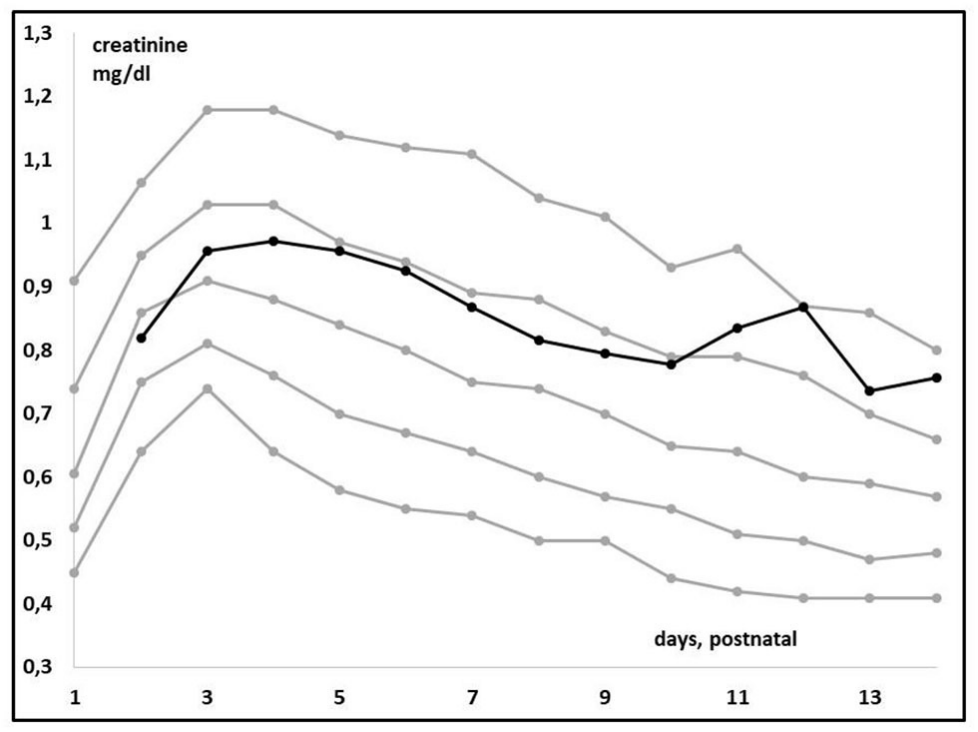
Trends in median creatinine values (enzymatic assay) in extreme low birth weight (ELBW) neonates when exposed to ibuprofen (*n* = 133, black line) compared to the reference centile trends (Table, gray lines) over time in the first 14 days of postnatal life (38).

**Figure 4:**
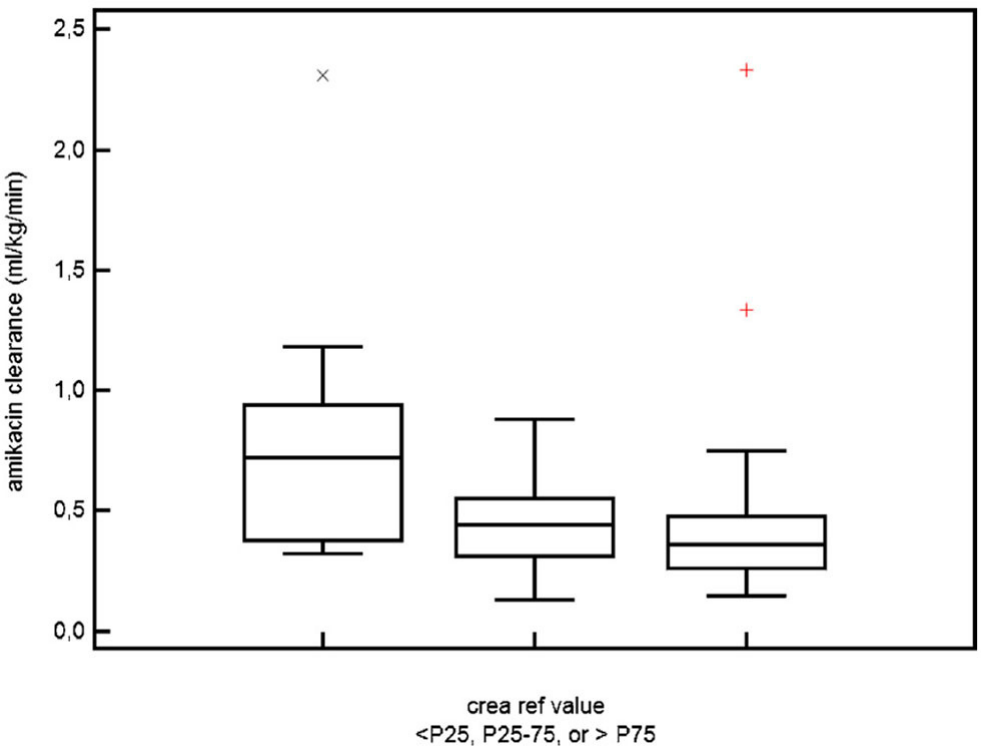
The relation between serum creatinine and amikacin clearance in ELBW neonates. Differences in amikacin clearance in ELBW neonates who had a serum creatinine reference value either <25th, between 25th and 75th, or >75th centile for ELBW neonates and for the specific postnatal day (38, 39, 41) (*permission for re-use of the figure has been granted, Rightslink*).

Plotting individual observations or S_cr_ trends over time in a single ELBW infant may facilitate recognition and quantification of an adverse drug event or may even facilitate precision pharmacotherapy, a concept somewhat similar to growth charts. To illustrate this, we compared mean S_cr_ in ibuprofen-exposed ELBWs in the earlier mentioned cohort to these centiles (38). Figure 3 (visual presentation of Table 2 data) illustrates the reference S_cr_ (gray lines) over postnatal age, with the plotted trend (black line) of median S_cr_ observed in ELBW neonates exposed to ibuprofen. A shift of about 1 standard deviation in S_cr_ 133 ibuprofen-exposed neonates is hereby observed (38, 39). On the other hand, we investigated how individual amikacin clearances are linked to S_cr_ centiles (38). For aminoglycosides like amikacin, there is a strong correlation between clearance and GFR, also in neonates (40). Consequently, GA and ibuprofen affect amikacin clearance in early neonatal life (41). Integration of S_cr_ centiles (<25th centile, 25–75th centile, or >75th centile) in this dataset further explained the individual amikacin clearance estimates (Figure 4) (38, 39). Along the same line, Cystatin C reference values (type of assay unclear, Modular Analytics ISE900 Analyzer, Germany) for ELBW (integrated in a cohort of very low birth weight, <1.5 kg) infants on day 1 and 3 [mean (SD) 1.77 (0.38) and 1.61 (0.37 mg/L, respectively)] have been suggested in the literature. In contrast to S_cr_ values, observations were independent of the GA (42).

### Key Messages

- Using the KDIGO-AKI definition results in an AKI incidence of about 50% in ELBW neonates. Similar to other populations, AKI is associated with increased mortality and morbidity.
- For pragmatic reasons, the criteria on urine output were converted to more extensive time intervals or weight trends.
- We suggest to use assay-specific S_cr_ centiles to better describe the normal postnatal trend and its variability. We illustrated that this approach facilitates recognition and quantification of adverse drug events (ibuprofen, Figure 3) or to explain individual amikacin clearance (Figure 4).

## Renal Injury Related to Asphyxia and Neonatal Encephalopathy

Perinatal asphyxia is a multi-organ disease, with moderate to severe encephalopathy as pivotal finding to initiate WBH in term neonates (4). This also includes AKI. As part of precision medicine, quantifying the incidence, extent, and variability of AKI and its covariates is relevant to tailor fluid administration and pharmacotherapy (4, 43). AKI has also been identified as prognostic factor for adverse neurological outcome and death (44, 45).

AKI occurs in neonates following perinatal asphyxia, but the incidence varies and in part depends on the case mix. In 36 neonates with asphyxia (Apgar score 5 min <7), AKI was documented in 1/11 (9%) with moderate and 12/25 (56%) with severe asphyxia (46). When we focus on WBH cases, AKI (according to the modified neonatal KDIGO criteria) was diagnosed in 39–42% (47, 48). Oliguria (<1 ml/kg/h for 12 h) was observed in 11% WBH neonates (49). In the most recent Cochrane meta-analysis, there was a trend to a lower incidence of renal impairment (urine output <0.5 ml/kg/h for ≥24 h + S_cr_ >1 mg/dl) for neonates undergoing WBH (38.5 vs. 45%, risk ratio and 95% CI, 0.87, 0.74–1.02), without effect on oliguria (<1 mg/kg/h, 23 vs. 24%) (50).

Table 3 provides an overview on S_cr_ observations (including assay) from day 1 to 10 as reported in cohorts of (pre)term asphyxia neonates (including criteria) in the era before WBH. Compared to the Gupta and Kaur cohorts, the other cohorts had more restrictive inclusion criteria and likely better reflect the neonates that currently qualify for WBH (19, 44, 51–54). These data remain valuable as some neonates may miss the 6-h therapeutic window or to subsequently compare such data with WBH-related observations. In these studies, different definitions for AKI or kidney failure were used (Table 3).

**Table 3:** Overview of serum creatinine observations (assay mentioned, mean, and standard deviation or interquartile range, all converted to mg/dl) on day 1, 2, or 3 and day 10 in term and preterm (light gray) asphyxiated neonates (criteria provided) and gestational age-matched controls (19, 44, 46, 51–54).

**Reference**	**Clinical features, asphyxia criteria**	**Assay**	**Day 1 asph.**	**Day 2/3 asph.**	**Day 10 asph.**	**Day 1 contr.**	**Day 2/3 contr.**	**Day 10 contr.**
Gupta, 2005 (51)	Apgar score 5 min <8, 59 term cases	Jaffe	n.a.	1.08 (0.49)	n.a.	n.a.	0.88 (0.26)	n.a.
Kaur, 2011 (46)	Apgar score 1 min <7, 36/2,196 cases, ≥34 weeks	Jaffe	0.92	0.95				
Sarafidis, 2012 (52)	Apgar score 5 min <7 + clinical HIE (any stage) 13 cases, 24 controls	Jaffe	1.32 (0.43)	1.57 (1.15)	0.73 (0.43)	1.02 (0.26)	0.76 (0.19)	0.69 (0.11)
Hadzimuratovic, 2014 (44)	Apgar score 5 min <7 + organ dysfunction + HIE 50 cases, 50 controls	Jaffe	n.a.	1.06 (0.40) (IQR)	n.a.	n.a.	0.65 (0.05) (IQR)	n.a.
Treiber, 2014 (19)	Apgar score 5 min <7 + organ dysfunction + HIE 50 cases, 50 controls	Jaffe	0.82 (0.17)	0.66 (0.18)	n.a.	0.71 (0.15)	0.57 (0.17)	n.a.
Gupta, 2005 (51)	Apgar score 5 min <8, 11 preterm cases	Jaffe	n.a.	1.34 (0.79)	n.a.	n.a.	0.83 (0.26)	n.a.
Song, 2017 (53)	Definition unclear, 34–37 weeks, 48 cases and 45 controls	?	0.74 (0.23)	1.07 (0.48)	n.a.	0.7 (0.28)	0.74 (0.29)	n.a.
Pan, 2018 (54)	Apgar score 5 min <4 + organ dysfunction + pH <7 (umbilical cord), <34 weeks, 71 cases, 70 controls	?	0.69 (0.15)	0.72 (0.14)	n.a.	0.72 (0.15)	0.74 (0.13)	n.a.

*? Stands for unknown, n.a. for not available*.

In the Gupta cohort, 47% of asphyxia neonates were classified as having “renal failure” [blood urea >40 mg/dl, S_cr_ >1 mg/dl (Jaffe) or oliguria (<0.5 ml/kg/h)] (51). Mean blood urea and S_cr_ on day 3 of postnatal life were significantly higher (+60 and 30%, respectively) in asphyxiated neonates, while the standard deviation (SD) for both biochemical markers doubled, indicating an increase in patient variability. This variability was in part explained by the HIE stage (mean [SD] S_cr_ for HIE stage 0, 1, 2, or 3 was 0.9 [0.2], 1.1 [0.4], 1.3 [0.8], 1.4 [0.6] mg/dl, respectively). Interestingly, the urine output was comparable between cases and controls while oliguria was rare (7/33 renal failure cases), while oliguric vs. non-oliguric renal failure was associated with higher mortality (43 vs. 8%). During follow-up, urine output normalized from day 4–6 onwards, urea and S_cr_ from day 7–9 onwards (51). In the more recently reported AWAKEN cohort (113 WBH neonates, AKI incidence 42%), oliguria was more commonly observed in AKI cases (isolated oliguria in 47, 26% mixed with S_cr_ thresholds, Table 1) (47). This confirms the complexity of AKI diagnosis, and the need to simultaneously assess both diuresis and S_cr_ or Cystatin C to tailor clinical care and pharmacotherapy. Kaur et al. reported on mean S_cr_ (Jaffe) values 24–36 h and 72–96 h in asphyxiated neonates (0.92 and 0.95 mg/dl), with significant differences between AKI and non-AKI neonates (1.49 vs. 0.8 mg/dl and 1.65 vs. 0.81 mg/dl) for both time intervals (46).

In the Sarafidis cohort, AKI (any S_cr_ > 1.5 mg/dl, or increase >0.3 mg/dl from day 1, Jaffe) was observed in 8/13 (61%). The Apgar score at 5 min was significantly lower in subsequent AKI neonates, while other indicators of asphyxia severity (inotropics, ventilation, anti-epileptic drugs, HIE moderate/severe, mortality) also associated with AKI (52). S_cr_ (day 1, 3, and 10) were significantly higher in asphyxiated neonates (1.32, 1.57, and 0.73 mg/dl) compared to controls (1.02, 0.76, and 0.69 mg/dl) with normalization on postnatal day 10. There is also a broader range (SD higher), reflecting higher inter-patient variability in renal impairment in asphyxia cases. In the Sarafidis cohort, several other biomarkers of renal GFR or tubular damage were also quantified. Serum Cystatin C (ELISA) was marginally increased only on day 1 in the asphyxiated neonates, while urine Cystatin C and Neutrophil Gelatinase-associated lipocalin were significantly increased in cases until day 10, suggesting earlier restoration of glomerular than tubular impairment (52). In the Hadzimuratovic cohort, S_cr_ (Jaffe) and Cystatin C (turbimetric assay) was significantly higher (63 and 49%, respectively) in asphyxia cases. In contrast to the findings of Sarafidis, both the absolute Cystatin C values and trends remained different, perhaps reflecting assay-related difference. The HIE stage (I vs. III) in part explained the S_cr_ variability on day 3. Finally, Treiber et al. also reported on renal biomarkers (S_cr_, Jaffe; Cystatin C, nephelometry) at birth (umbilical cord) and on day 3 in a cohort of asphyxia neonates. Interestingly, S_cr_ and Cystatin C were significantly higher from delivery onwards. Umbilical Cystatin C was the most sensitive marker of asphyxia (receiver operating characteristic curve = 0.918) (Table 3).

A specific focus on preterm neonates is warranted. These patients do not qualify for WBH but can still display asphyxia-related AKI (Table 3). Gupta et al. (51) documented that the mean S_cr_ is significantly higher (+60%) in asphyxiated (Apgar score 5 min <8) preterms. Song et al. (53) reported on S_cr_ (assay unclear) values in near-term (34–37 GA) asphyxia (definition unclear, *n* = 48) and age-matched controls, and observed a significant decrease (−20%) in eGFR in asphyxia cases. Pan et al. included 71 preterms (<34 weeks) with asphyxia (pH <7 + Apgar at 5 min <4 + multi organ dysfunction) and 70 preterm controls, and collected samples at 24, 48, and 96 h. S_cr_ was significantly higher at 96 h (83.5 vs. 62.9 μmol/l) and eGFR was consistently lower at 24 and 48 h (−30 and −20%, respectively) (54). Cystatin C (PENIA) had good distinguishability between asphyxiated and non-asphyxiated preterms, irrespective (<28, 28–32, or ≥32 weeks subgroups) of GA, and further discriminated between mild, moderate, and severe asphyxia. In contrast, S_cr_ (assay unclear) was not discriminative (55).

AKI affects the renal and non-renal outcome in asphyxia neonates. AKI is associated with a 4.6-fold higher mortality risk in the AWAKEN study, and this higher mortality risk also holds true for post-asphyxiated neonates with AKI (29, 51). When considering the renal outcome after discharge, Hadzimuratovic et al. (44) and Gupta et al. (51) reported on normalization of renal findings at 1 and 6 months, respectively in asphyxia cases (44, 51). AKI also associates with non-renal outcome, like prolonged hospital stay (48), prolonged mechanical ventilation (47), or abnormal brain imaging findings (73 vs. 46%) at the end of the first week of life (56). Post-asphyxial renal injury (urine output + S_cr_) was a prognostic factor for neurological outcome at the end of the 1st year of life (44). However, the absence of AKI neither guarantees a positive outcome (45).

Once AKI has been identified, precision medicine involves fluid and electrolyte management, drug choice (avoid nephrotoxic drugs, potential nephro-protective interventions), and for those drugs, dose selection.

In the earlier mentioned AWAKEN study, the daily %-weight change from birth weight in the 1st week of postnatal life was used to reflect the fluid balance in 645 critically ill term neonates. A higher peak fluid balance and higher fluid balance over the first week of life were independently associated with mechanical ventilation on day 7. A negative fluid balance was observed in 53% of neonates (21 vs. 41% in ventilated vs. non-ventilated neonates). Those with AKI had a consistently higher fluid balance throughout the 1st week of life (36). Suggested nephro-protective interventions include “low dose dopamine” or methylxanthines, like theophylline (57). At present, there is no robust evidence for the use of low dose dopamine to protect kidney function, while there is meta-analytic evidence that prophylactic theophylline (single intravenous dose, 5 mg/kg) results in a significant lower AKI incidence (OR 0.24) in asphyxiated neonates (58). Despite the evidence, neonatologists remain reluctant to administer theophylline as this increases metabolic activity of the brain, while hypothermia and sedation are used to reduce metabolic (cerebral) activity.

With respect to precision pharmacotherapy, it is important to realize that the mean difference in S_cr_ or eGFR between asphyxiated neonates (either or not undergoing WBH) compared to term controls is clinical significant (−40 to −50%) and further adds to maturational (weight, postnatal) changes. This is reflected by the impact of asphyxia on drugs exclusively cleared by renal elimination (4). To illustrate this, amikacin clearance trends in early neonatal life based on pooling of reported datasets were plotted (**Figure 6**). There is a maturational trend in clearance, related to birth weight and postnatal age (day 1, 2, 3, 4, as indicated by different colors) compared to a subgroup of WBH neonates. These differences indicate mean differences in clearance, but do not cover the additional unexplained between-individual variability (59). Essentially, there is a shift in the Gauss curve for S_cr_ or eGFR toward renal impairment, but mean differences do not fully cover the between individual variability. For this type of drugs, this means that the time interval between consecutive administrations should be extended (in general from 24 to 36 h, so compensating for the 40–50% decrease in clearance) but therapeutic drug monitoring remains compulsory as there still will be toxic trough levels in 14–25% of cases (59).

### Key Messages

**Figure 5:**
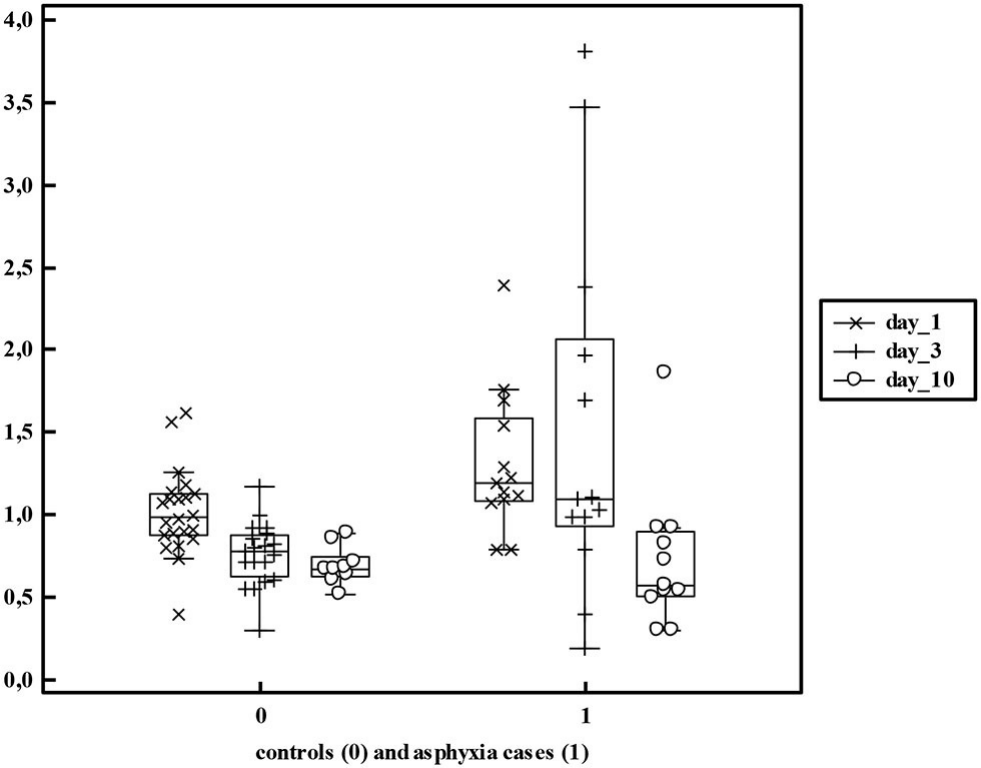
Serum creatinine values in non-asphyxiated (*n* = 24) and asphyxiated neonates (*n* = 13) as reported by Sarafidis et al. (52) reflecting both the mean differences and additional variability (>5 fold) within these cohorts.

**Figure 6:**
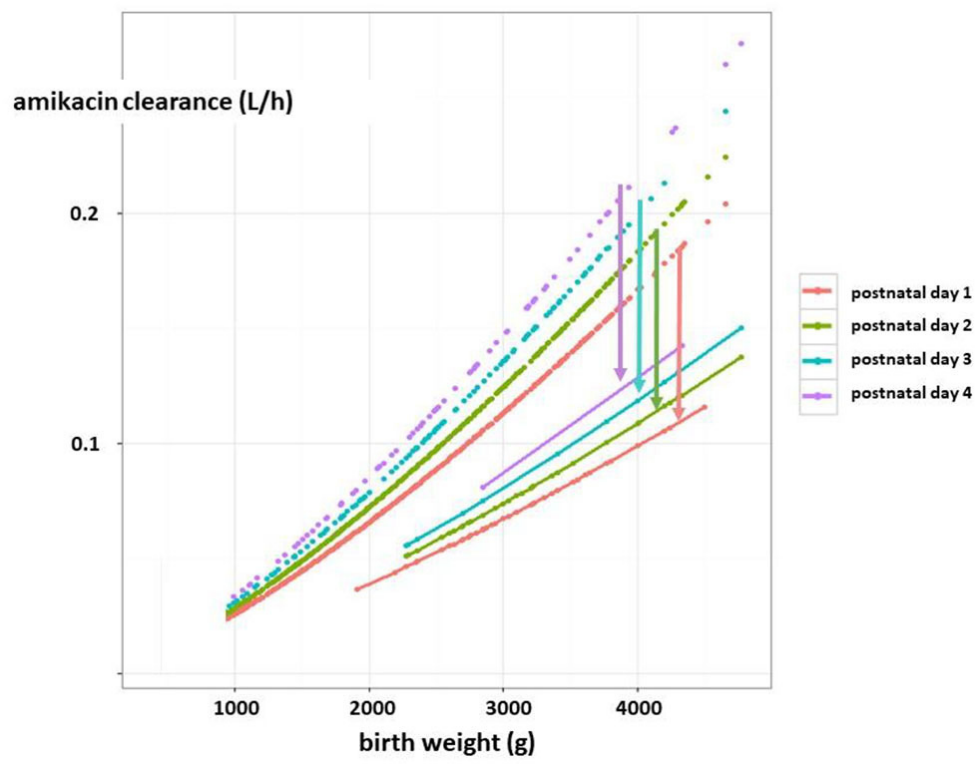
Estimates of amikacin clearance (l/h) trends in early neonatal life based on pooling of reported datasets (dashed lines). There is a maturational trend in clearance, related to birth weight (g) and postnatal age (day 1, 2, 3, 4, as reflected by the colors) compared to a subgroup of term neonates undergoing therapeutic hypothermia as treatment for perinatal asphyxia (solid lines). The arrows indicate the difference in clearance between both cohorts for the respective postnatal age (in days) (4, 59) [*this figure has earlier been published in reference* *(**4**), permission for re-use has been granted, first author Anne Smits*].

- Oliguria is a specific indicator of AKI, but the majority of AKI cases are non-oliguric. Assessment of the fluid balance is an alternative. This confirms the complexity of AKI diagnosis, and the need to simultaneously assess both diuresis and S_cr_ or Cystatin C to tailor clinical care and pharmacotherapy.
- Asphyxia with WBH results in a clinical significant—often transient—mean decrease in eGFR (−40 to −50%), with GA and HIE stage as additional covariates (Table 3).
- This mean decrease in GFR affects renal drug elimination. However, there is still large (unexplained) inter-individual variability in GFR (mean vs. SD) and renal drug clearance in asphyxiated neonates (Figures 5, 6).

## Renal Precision as Crucial Part of Contemporary Neonatal Precision Medicine

Using ELBW and asphyxia as case examples, we illustrated that kidney function and AKI are relevant to contemporary neonatal care. In ELBW, AKI is associated with increased mortality and morbidity while S_cr_ centiles were used to recognize and quantify adverse drug events and to explain individual amikacin clearance. Asphyxia associated AKI affects mortality and morbidity. WBH is associated with a significant mean (−40 to −50%) GFR decrease. Of relevance, there is still important variability in GFR decrease around this mean decrease, so that the mean decrease does not predict well the GFR decrease in an individual neonate. Although these data strongly suggest that better integration of renal precision is important to improve contemporary neonatal care, the AWAKEN study showed that the median number of S_cr_ measurements was ≤3 and ≤5/patient in 10 and 15/24 of the units, suggesting that further improvements can be made (29). There are some elegant illustrations on how these improvements can be implemented and how a focus on renal aspect indeed improves neonatal care.

In the Baby NINJA study, 476 individual events of high-risk nephrotoxic drug exposure were observed. During these events, a daily S_cr_ was obtained until 2 days after exposure or after end of AKI. Within this framework, there was a reduction in exposure (16.4 to 9.6/1,000 patient days), a reduction in drug-associated AKI (30.9 to 11%), and in AKI intensity (9.1 to 2.9/100 susceptible patient days) (60). Implementation of AKI guidelines in a single NICU resulted in improvements in recognition, diagnosis, and subsequent follow-up of AKI (61). This matters, as in ELBW infants, exposure to nephrotoxic drugs is common (87%) with gentamicin (86%), indomethacin (43%), and vancomycin (25%) as most commonly administered drugs (62). It is hereby important to highlight that follow-up of these populations remains important after hospital discharge as there are concerns on the long-term renal outcome, most pronounced in former ELBW cases (63). Research should focus on perinatal risk factors associated with impaired GFR in long-term outcome studies, but is hampered by single center cohorts, small samples sizes, and heterogeneity of GFR assessment tools (64). The diagnosis of AKI remains complex with integrated assessment both of diuresis and S_cr_ or Cystatin C to tailor clinical care and pharmacotherapy. We therefore state that further integration of renal (patho)physiology into neonatal precision medicine and pharmacotherapy may not only result in better short-term outcome but also may have impact throughout pediatric life and beyond.

## Author Contributions

KA initiated the project. AS and KS hereby had a specific focus on the section on acute kidney injury following perinatal asphyxia. TD and JA had a specific focus on the section of acute kidney injury. EL and DM provided crucial nephrologic expertise on these sections and on the introduction and discussion section. All authors approved the final version of the paper.

## Conflict of Interest

The authors declare that the research was conducted in the absence of any commercial or financial relationships that could be construed as a potential conflict of interest.
